# Molecular Mechanism Underlying the Interaction of Typical Sac10b Family Proteins with DNA

**DOI:** 10.1371/journal.pone.0034986

**Published:** 2012-04-12

**Authors:** Yan-Feng Liu, Nan Zhang, Xi Liu, Xinquan Wang, Zhi-Xin Wang, Yuanyuan Chen, Hong-Wei Yao, Meng Ge, Xian-Ming Pan

**Affiliations:** 1 Ministry of Education Key Laboratory of Bioinformatics, School of Life Sciences, Tsinghua University, Beijing, People's Republic of China; 2 National Laboratory of Biomacromolecules, Institute of Biophysics, Chinese Academy of Sciences, Beijing, People's Republic of China; Institute of Molecular and Cell Biology, Singapore

## Abstract

The Sac10b protein family is regarded as a family of DNA-binding proteins that is highly conserved and widely distributed within the archaea. Sac10b family members are typically small basic dimeric proteins that bind to DNA with cooperativity and no sequence specificity and are capable of constraining DNA negative supercoils, protecting DNA from Dnase I digestion, and do not compact DNA obviously. However, a detailed understanding of the structural basis of the interaction of Sac10b family proteins with DNA is still lacking. Here, we determined the crystal structure of Mth10b, an atypical member of the Sac10b family from *Methanobacterium thermoautotrophicum ΔH*, at 2.2 Å. Unlike typical Sac10b family proteins, Mth10b is an acidic protein and binds to neither DNA nor RNA. The overall structure of Mth10b displays high similarity to its homologs, but three pairs of conserved positively charged residues located at the presumed DNA-binding surface are substituted by non-charged residues in Mth10b. Through amino acids interchanges, the DNA-binding ability of Mth10b was restored successfully, whereas the DNA-binding ability of Sso10b, a typical Sac10b family member, was weakened greatly. Based on these results, we propose a model describing the molecular mechanism underlying the interactions of typical Sac10b family proteins with DNA that explains all the characteristics of the interactions between typical Sac10b family members and DNA.

## Introduction

The Sac10b protein family, also named Alba, is generally regarded as a family of DNA-binding proteins that are highly conserved and widely distributed within the archaea [1]–[4]. Typical Sac10b family members, such as Sac10b from *Sulfolobus acidocaldarius* [5]–[7], Ssh10b from *S. shibatae* [8]–[12], Sso10b and Sso10b2 from *S. solfataricus* [13]–[18], Mja10b from *Methanocaldococcus jannaschii* [19], and Afu10b from *Archaeoglobus fulgidus* [20], are small basic dimeric proteins which bind to DNA with cooperativity and no apparent sequence specificity. They are capable of constraining negative supercoils, protecting DNA from Dnase I digestion, and do not compact DNA obviously *in vitro*.

The crystal structure of Sso10b was solved in 2002 [14]; Sso10b belongs to the mixed class (α/β) of proteins and resembles the C-terminal domain of the bacterial translation initiation factor IF3. The overall structure of the Sso10b dimer appears to be like a body with two long, outstretched β-hairpin arms. Sso10b is acetylated at Lys16 *in vivo*, resulting in a several-fold reduction in its nucleic acid-binding affinity [13]–[15]. Results from DAPI dye displacement assays suggest that Sso10b binds to DNA in its minor grooves. Based on these reports, Wardleworth *et al.* first proposed a model for the interaction of Sso10b with the DNA duplex [14]. According to this model, the two flexible β-hairpin loops interact with equivalent minor groove regions and allow the highly basic central body to make DNA contacts in the major groove regions. In following years, several similar models describing the interactions of these proteins with DNA were suggested in succession alongside the publication of the structures of several other Sac10b members, including Sso10b2 from *S. solfataricus* [17], [18], Ssh10b from *S. shibatae* [10], Mja10b from *M. jannaschii* [19], Afu10b from *A. fulgidus* [20], Ape10b2 from *A. pernix K1* [21], and Pho10b from *P. horikoshii OT3* [22]. However, a detailed understanding of the structural basis of the interaction of Sac10b family proteins with DNA is still lacking.

**Figure 1:**
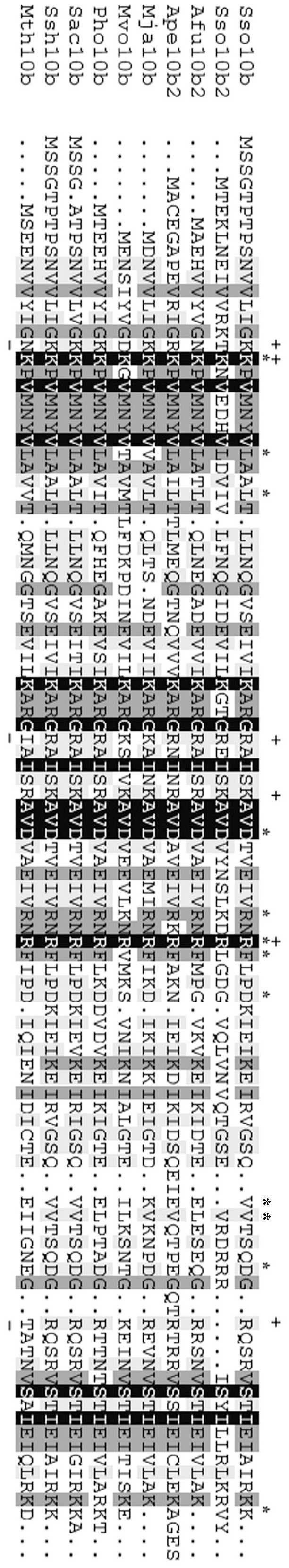
Sequence alignment of Mth10b and other typical members of the Sac10b protein family: Sso (*Sulfolobus solfataricus*), Afu (*Archaeoglobus fulgidus*), Ape (*Aeropyrum pernix K1*), Mja (*Methanocaldococcus jannaschii*), Mvo (Methanococcus voltae), Pho (*Pyrococcus horikoshii*), Sac (*Sulfolobus acidocaldarius*), Ssh (*Sulfolobus shibatae*), and Mth (*Methanobacterium thermoautotrophicum*). The six conserved positively-charged residues located at the DNA-binding surface are marked at the top with plus signs (+). The three substituted neutral residues in Mth10b are marked at the bottom with minus signs (−). The residues involved in the interactions formed between two adjacent Sso10b dimers along the axis of the DNA are marked at the top with asterisks (*).

Recently, we identified an atypical member of the Sac10b family, Mth10b from *M. thermoautotrophicum ΔH* [23], whose amino acid sequence shares high similarity with its archaeal homologs (Figure 1). However, unlike typical Sac10b family proteins, Mth10b is an acidic protein which contains 13 acidic amino acid residues and only 8 basic residues, and its potential isoelectric point is only 4.56 [23]. Biochemical characterization suggests that Mth10b should be similar to typical Sac10b family proteins with respect to its secondary and tertiary structure and in its preferred oligomeric forms [23]. However, electrophoretic mobility shift analysis (EMSA) showed that Mth10b does not bind to either DNA or RNA [23]. We reasoned that understanding the molecular basis underlying the functional deviation of Mth10b from the other family members may provide new insights into the mechanism underlying the interaction of typical Sac10b family members with DNA. In the present work, we determined the crystal structure of Mth10b at a resolution of 2.2 Å (PDB code: 3TOE). While the overall structure displays significant similarity to the structures of other reported Sac10b family members, three pairs of conserved positively-charged residues located at the presumed DNA-binding surface are substituted by non-charged residues in Mth10b. When these non-charged residues of Mth10b were altered to the corresponding positively-charged residues by site-directed mutagenesis, the DNA-binding ability of Mth10b was successfully restored. Conversely, when the positively-charged residues of Sso10b, a typical Sac10b family member, were altered to the corresponding non-charged residues, its DNA-binding ability decreased greatly. Based on these results, we propose a novel model describing the molecular mechanism underlying the interactions of typical Sac10b family proteins with DNA which explains all the characteristics of the interactions between typical Sac10b family proteins and DNA.

## Results

### Overall structure of Mth10b

**Figure 2:**
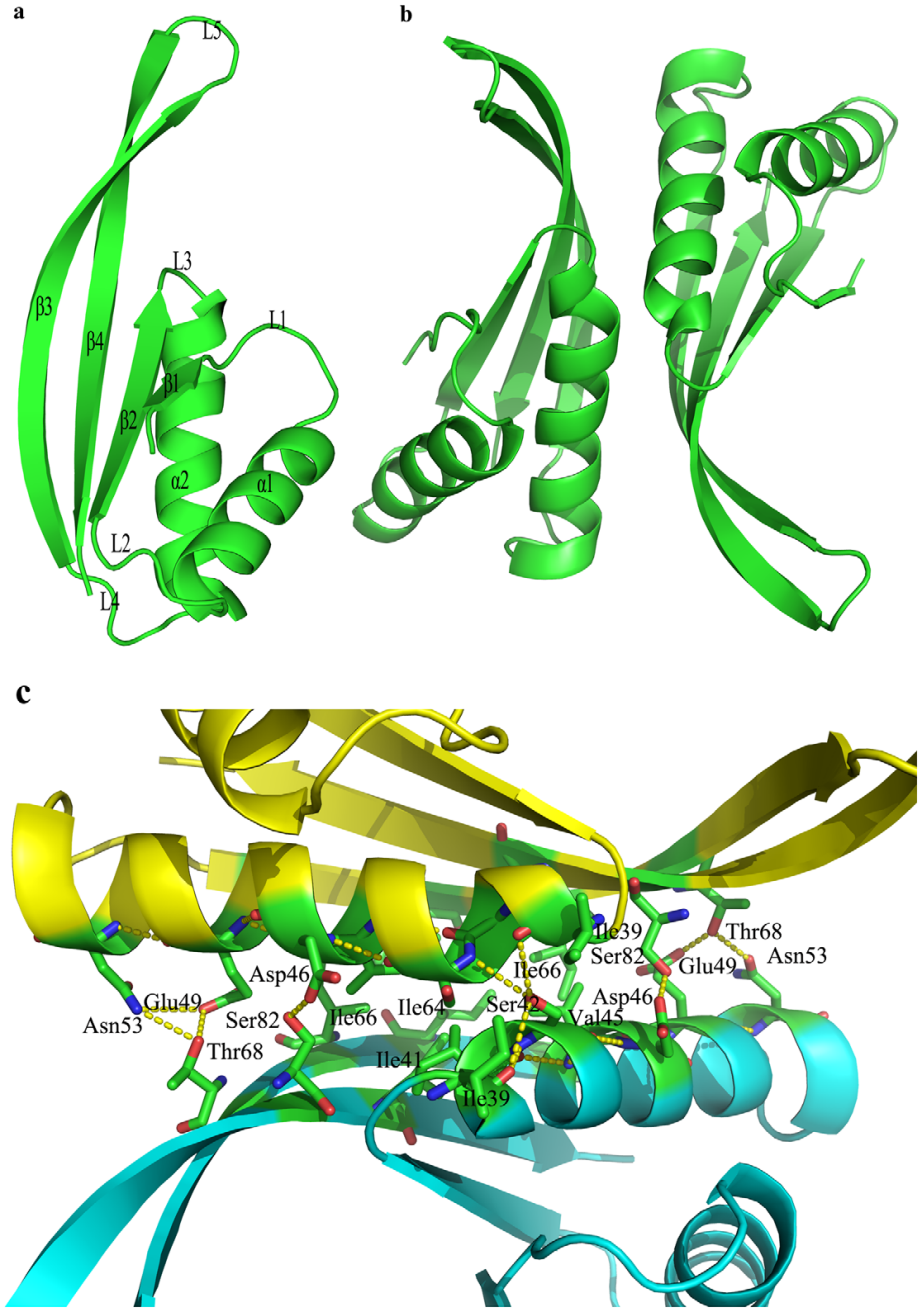
Crystal structure of Mth10b. (**a**) Ribbon diagram of the Mth10b monomer. The helices, strands and loops are labeled as α1 to α2, β1 to β4, and L1 to L5 respectively. (**b**) Ribbon diagram of the Mth10b dimer. (**c**) View of the Mth10b homodimer interface. Residues involved in dimerization are indicated. Hydrogen bonds formed between side chains are also indicated with dashed lines.

The crystal structure of the Mth10b monomer is of mixed α/β type, and consists of two α-helices and four β-strands with a topology of β_1_-α_1_-β_2_-α_2_-β_3_-β_4_. As shown in Figure 2a, these two α-helices and four β-strands are connected through five different loops. The strands β_1_, β_2_ and β_4_ are parallel, while strand β_3_ is antiparallel to the other strands. This structure model covers 86 of the total 91 residues, and no electron density was observed for the four N-terminal residues or for C-terminal Asp91. The monomer structure is asymmetric with approximate dimensions of 47×27×21 Å. Two adjacent asymmetric Mth10b monomers interact with each other side-by-side along the long dimension of the monomer, forming a dimer with a crystallographic two-fold axis perpendicular to the long dimension of the dimer (Figure 2b). The overall structure of the Mth10b dimer resembles a body with two long, extending, flexible β-hairpin arms, agreeing well with the dimeric structures reported for typical members of the Sac10b family. Helix α_2_, and strands β_3_ and β_4_ are involved in dimerization (Figure 2c), through hydrophobic interactions as well as hydrogen bonding. Hydrophobic residues Ile39, Ile41, Val45, Ile61, Ile64, Ile66 and Ile84 from each monomer form a hydrophobic surface, and polar residues Ser42, Asp46, Glu49, Asn53, Thr68 and Ser82 also contribute to dimer stabilization by forming hydrogen bonds with their neighboring residues. Residues involved in dimerization are almost all highly conserved across the Sac10b protein family (Figure 1). In addition, each monomer in the Mth10b structure contains four salt bridges: Glu31-Arg89, Lys35-Glu85, Arg37-Glu69, and Glu49-Arg52. These salt bridges are all located on the convex surface of the dimer and may play an important role in bracing and stabilizing the structure, as salt bridge interactions are considered critical for the stability of thermophilic proteins [24], [25].

### Structure comparison

**Figure 3:**
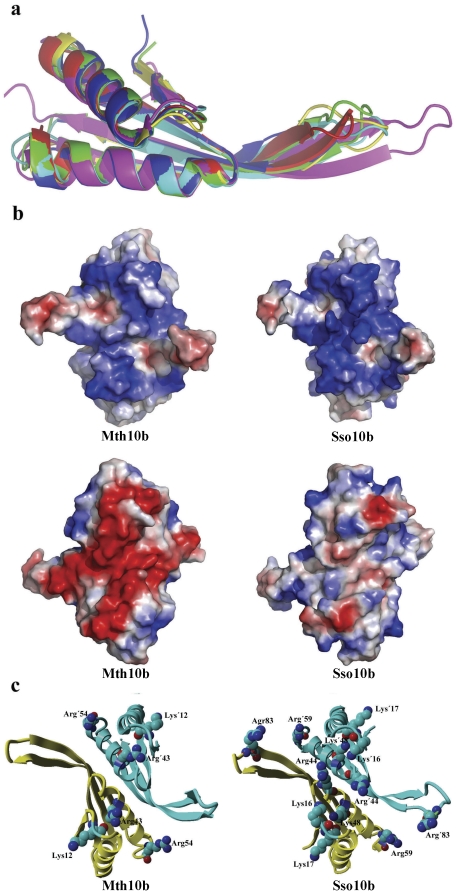
Structural comparison of Mth10b with its archaeal homologs. (**a**) Superposition of the ribbon diagrams of Mth10b and its homologs. Mth10b, Sso10b (1H0Y), Mja10b (1NH9), Afu10b (1NFJ), Ape10b2 (2H9U) and pho10b (2Z7C) are colored in red, green, blue, yellow, magenta and cyan respectively. (**b**) Comparison of the surface electrostatic potential of Mth10b and Sso10b (1h0x). The electrostatic potential was calculated using PYMOL. The upper left panel and the upper right panel represent the concave surface electrostatic potential of Mth10b and Sso10b respectively. The lower left panel and the lower right panel represent the convex surface electrostatic potential of Mth10b and Sso10b respectively. (**c**) Comparison of the positively-charged residues located at the concave surface of Mth10b and Sso10b: the left panel is Mth10b; the right panel is Ssh10b. Residues with (or without) a prime belong to the same monomer.

The overall structure of Mth10b shares high similarity with its archaeal homologs. Superposition of the structure of Mth10b on those of typical family members (Figure 3a), including Sso10b (1H0Y) and Sso10b2 (1UDV) from *S. solfataricus*, Mja10b (1NH9) from *M. jannaschii*, Afu10b (1NFJ) from *A. fulgidus*, Ape10b2 (2H9U) from *A. K1*, and Pho10b (2Z7C) from *P. horikoshii OT3*, gave rmsd values of 0.624 Å, 0.803 Å, 0.526 Å, 0.687 Å, 1.442 Å and 0.822 Å, respectively. The main differences among these structures occur at the tips of the outstretched β-hairpin arms (Figure 3a), suggesting that the loops between strands β3 and β4 may be inherently flexible. The sequences of this region are not conserved.

Figure 3b shows the surface electrostatic potential map of Mth10b and the typical Sac10b family member Sso10b. Calculation of the surface electrostatic potential reveals that Mth10b has an extremely uneven charge distribution. Though Mth10b is an acidic protein, the concave surface of the molecule corresponding to the DNA-binding surface in Sso10b is dominated by positively-charged residues, but its positive electrostatic potential is much lower than that of Sso10b (Figure 3b). Interestingly, the electrostatic potential of the convex surface is extremely negative, while the corresponding surface in Sso10b is almost neutral (Figure 3b). Further analysis reveals that the positive electrostatic potential of the DNA-binding surface in Sso10b mainly results from six pairs of positively-charged residues, namely Lys16, Lys17, Arg44, Lys48, Arg59, and Arg83 in each monomer, located at the concave surface (Figure 3c, right) of the homodimer. These residues are highly conserved in the Sac10b family (Figure 1). However, there are only three pairs of positively-charged residues, namely Lys12, Arg43 and Arg54 in each monomer, located on the corresponding concave surface of Mth10b (Figure 3c, left). The other three pairs of conserved positively-charged residues, namely Lys16, Arg44 and Arg83 in each Sso10b monomer, are substituted, respectively, in Mth10b by neutral residues Asn11, Ile39 and Thr77 (Figure 1), suggesting that these three pairs of residues should be key to the interaction between typical Sac10b family proteins and DNA.

### Mutant protein expression and purification

**Figure 4:**
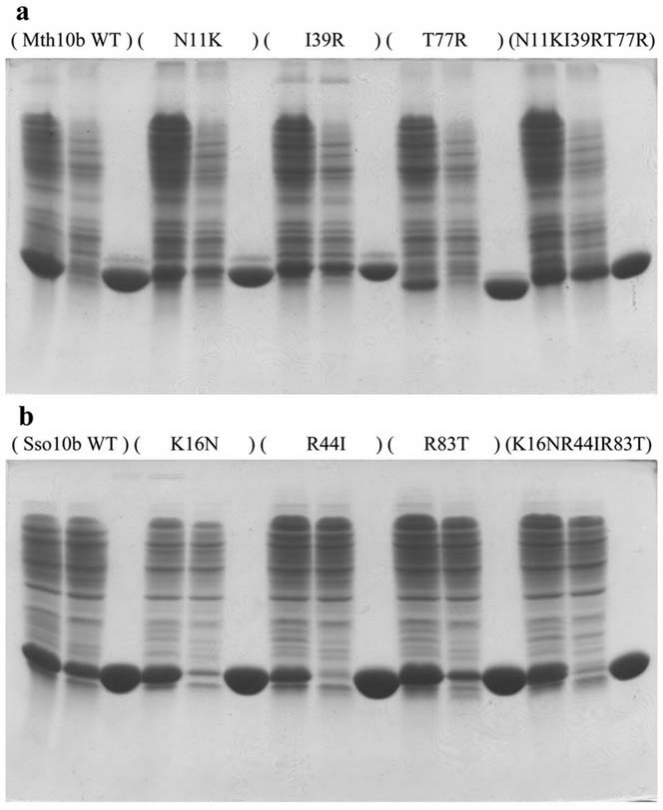
SDS-PAGE of protein expression and purification. From left to right, every three consecutive lanes represent a protein, and the protein name is given at the top of the lane. (**a**) Wild type Mth10b and its mutants. For each protein, from left to right, the three lanes represent the sample before the Q Sepharose Fast Flow column, the pass-through sample and the final purified protein. (**b**) Wild type Sso10b and its mutants. For each protein, from left to right, the three lanes represent the sample before the SP Sepharose Fast Flow column, the pass-through sample and the final purified protein.

**Figure 5:**
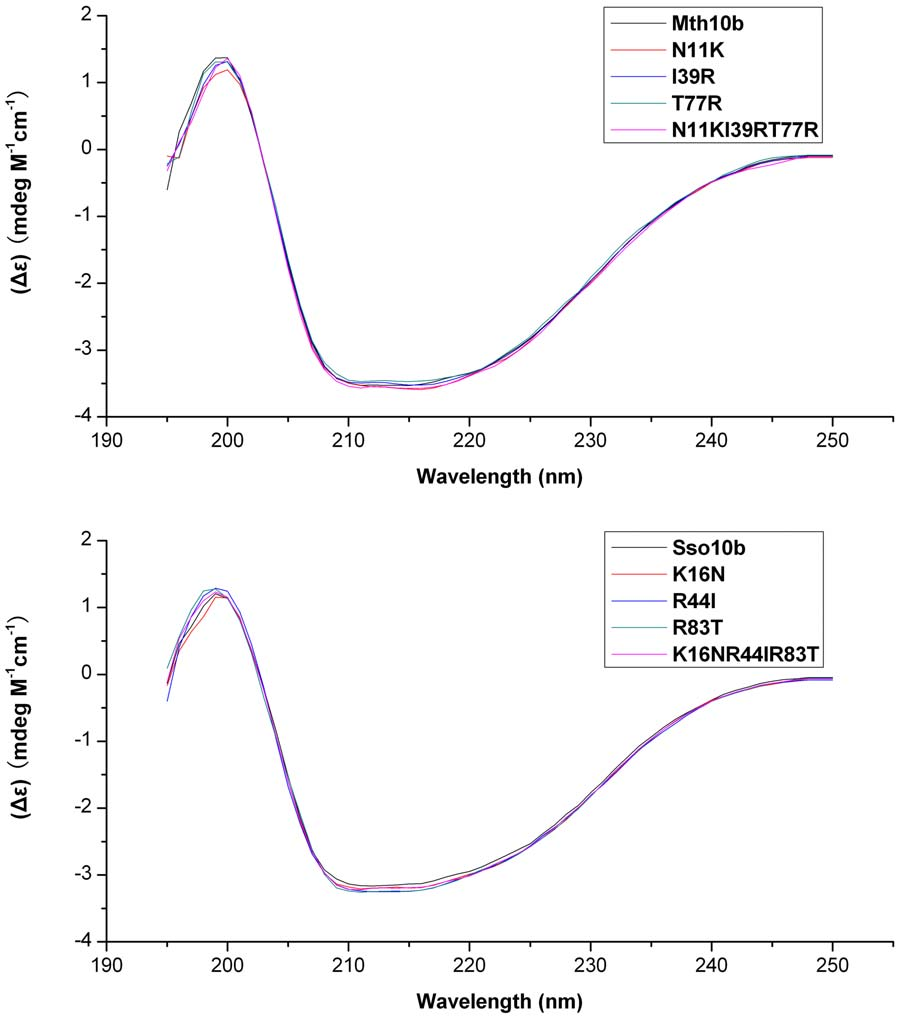
CD spectra (a) Mth10b and its variants; (b) Sso10b and its variants. CD measurements were performed as described previously [23].

Mutants of Mth10b and Sso10b were constructed by site-directed mutagenesis. Mutant proteins, along with wild type Mth10b and wild type Sso10b, were expressed in E. *coli* BL21 (DE3) and purified to homogeneity. Protein purity was higher than 95% as confirmed by 15% SDS-PAGE (Figure 4). CD spectra of Mth10b and Sso10b agree well with previous reports [23], [24], indicating that they have a well-folded structure (Figure 5). Moreover, CD spectra of the mutants and their wild-types are highly superimposable, indicating that amino acid residue replacements do not influence their structure (Figure 5).

During protein purification, two interesting phenomena were found. Though the isoelectric points of the Mth10b mutants increased on amino acid substitution, when wild type Mth10b and its mutants were purified by an anion exchange column, their retention times were almost the same. This phenomenon suggests that these proteins bound to the anion exchange column at their extremely negatively-charged convex surface (Figure 3b), when mutations were introduced in their concave surface. Moreover, when wild type Mth10b and the single mutant T77R were purified on a Q Sepharose Fast Flow column, the proteins in the samples were almost totally captured by the column; while when the other two single mutants N11K and I39R, and the triple mutant N11KI39RT77R were purified, a significant amount of protein remained in the pass-through samples (Figure 4a). Especially in the case of the triple mutant, only a small fraction of the protein was captured by the column. In order to obtain sufficient protein, the pass-through sample was repeatedly purified with the same column several times. Considering that the retention times of these proteins are almost the same, this phenomenon may result because genomic DNA in the sample competes with the column for interaction with these proteins, suggesting that the DNA-binding ability of Mth10b may be restored in the two single mutants (N11K and I39R) and the triple mutant to various extents. Conversely, when wild type Sso10b and its mutants were purified by a cation exchange column, their retention times were shorter as their isoelectric points decreased, suggesting that these proteins bound to the cation exchange column at their extremely positively-charged concave surface (Figure 3b), where mutations were introduced. When wild type Sso10b was purified by a Source 30S column, only a fraction of the protein was captured. However, the amount of protein remaining in the pass-through sample for the single mutant R83T was notably decreased (Figure 4). Moreover, there was no notable protein remaining in pass-through samples of the two other single variants (K16N and R44I) or the triple variant K16NR44IR83T (Figure 4b). These results suggest that the DNA-binding ability of these Sso10b mutants is weakened to various extents.

### Biosensor-surface plasmon resonance (SPR) determination of equilibrium dissociation constants

SPR measurements were employed to gain insights into the binding properties of Mth10b, Sso10b and their mutants. A 110 bp biotinylated DNA duplex (76.4 RU) was immobilized on a SA sensor chip. Increasing concentrations of purified proteins were injected and the interactions between these proteins and the DNA duplex were measured in different running buffers.

**Figure 6:**
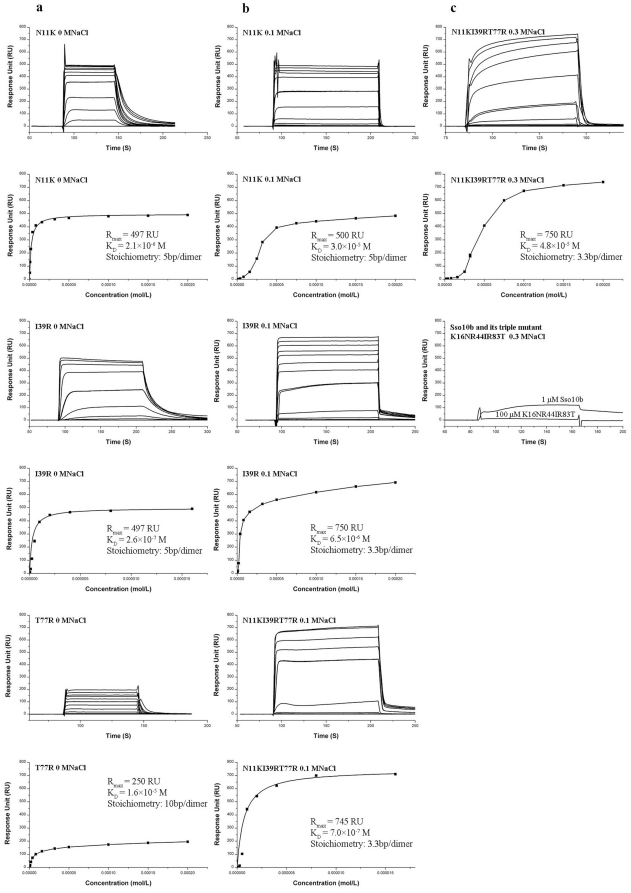
SPR measurements in basic running buffer without NaCl (a), containing 0.1 M NaCl (b) and containing 0.3 M NaCl (c). The names of the tested proteins, their K_D_, R_max_ and saturation binding stoichiometry values are marked on each panel.

When the basic running buffer (20 mM Tris, 1 mM EDTA, 0.005% Tween20/pH 7.5) without NaCl was used, no interaction was observed between wild type Mth10b and the DNA duplex, agreeing well with EMSA results [23]. Measurements for the three single mutants of Mth10b (Figure 6a) gave apparent dissociation constant (K_D_) values of 2.1×10^−6^ M, 2.6×10^−7^ M and 1.6×10^−5^ M for N11K, I39R and T77R, respectively. Slight cooperativity was observed in these three mutants (Figure 6a). These results suggest that the DNA-binding ability of these three single mutants was restored to various extents. Measurements for the triple mutant of Mth10b, and for Sso10b and its mutants, in the basic running buffer could not be carried out due to high non-specific interactions between proteins and the dextran matrix of the chip.

When a basic running buffer containing 0.1 M NaCl was used, there was also no signal response observed for wild type Mth10b. The signal response of the single mutant T77R was low (only 18.0 RU even at 100 µM), while measurements for the other three mutants of Mth10b, N11K, I39R and the triple mutant N11KI39RT77R, gave K_D_ values of 3.0×10^−5^ M, 6.5×10^−6^ M and 7.0×10^−7^ M, respectively (Figure 6b). Relative to measurements in the basic running buffer without NaCl, the DNA affinity of N11K, and I39R decreased about 15- and 25-fold respectively, and the DNA affinity of T77R decreased to a hardly measurable level. These results may be due to soluble salt ions partially shielding the electrostatic interactions between these proteins and the DNA duplex. The DNA affinity of the triple mutant N11KI39RT77R was markedly stronger than that of the three single mutants, indicating that the contribution of these three positively-charged residues to DNA-binding ability is additive. While slight cooperativity was observed in the cases of I39R and N11KI39RT77R, cooperativity was marked for N11K (Figure 6b). Measurements for Sso10b and its mutants in the basic running buffer containing 0.1 M NaCl could not be performed due to high non-specific interactions.

In order to decrease non-specific interactions, the salt concentration in the running buffer was further increased to 0.3 M NaCl. Under this condition, only the K_D_ value of the triple mutant N11KI39RT77R (Figure 6c) could be measured, and its DNA-binding ability decreased about 70-fold relative to that measured in the basic running buffer containing 0.1 M NaCl. Moreover, the triple mutant bound to DNA with high cooperativity (Figure 6c). Non-specific interactions between proteins and the dextran matrix of the chip were still marked in the cases of Sso10b and its mutants, and thus quantitative determination of the K_D_ values of these proteins was still beyond reach. As an alternative, the DNA-binding ability of wild type Sso10b and its triple mutant K16NR44IR83T were compared qualitatively in the basic running buffer containing 0.3 M NaCl. As shown in Figure 6c, injection of 1 µM wild type Sso10b produced a signal response of about 120 RU, but injection of 100 µM K16NR44IR83T produced almost no response. Though high non-specific interactions between proteins and the chip may partially mask the true situation, based on these results we can conclude roughly that, relative to the wild type, the DNA-binding ability of the Sso10b mutant K16NR44IR83T decreased greatly, and may have even been lost totally, indicating these three positively-charged residues are key for the DNA-binding ability of typical Sac10b family members.

Results of SPR measurements for the triple mutant of Mth10b, N11KI39RT77R (Figure 6), suggest a saturation binding stoichiometry of about 3.3 bp per dimer, a little lower than the ∼5 bp previously reported by Wardleworth *et al.* [14], but agreeing well with results from an early report [6]. However, the situation was more complicated for the three single mutants: N11K and T77R showed saturation binding stoichiometries of about 5 bp and 10 bp per dimer, respectively, and I39R displayed a saturation binding stoichiometry of about 5 bp per dimer in the basic running buffer, but about 3.3 bp per dimer in the basic running buffer containing 0.1 M NaCl. These results indicate that these mutants have different binding modes.

### Analysis of protecting DNA from Dnase I digestion

**Figure 7:**
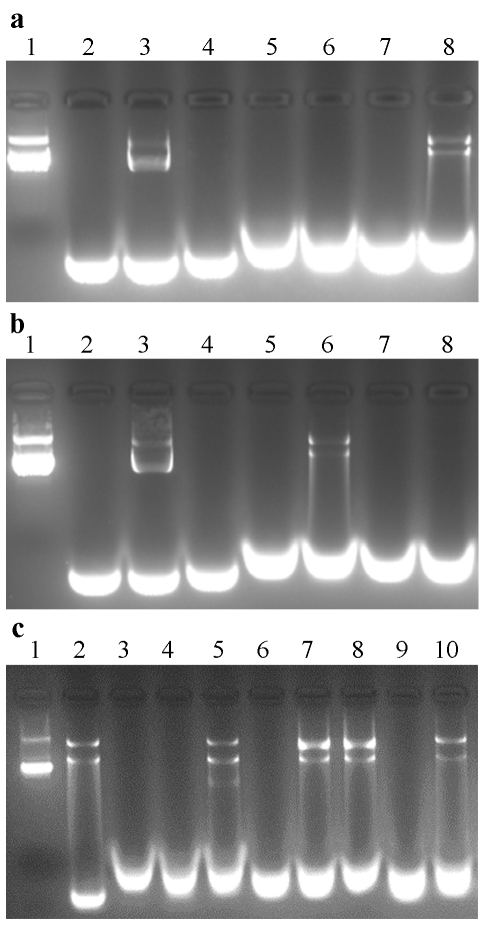
Protection of DNA from Dnase I digestion. (**a**) Sso10b and its triple mutant. and (**b**) Mth10b and its triple mutant: Lane 1, plasmid alone; lane 2, 1% SDS alone; lane 3, plasmid with addition of SDS; lane 4, plasmid digested with 0.5 U Dnase I before addition of SDS; lane 5, triple mutant incubated with 0.5 U Dnase I before addition of SDS; lane 6, DNA incubated with the triple mutant then digested with 0.5 U Dnase I before addition of SDS; lane 7, wild type incubated with 0.5 U Dnase I before addition of SDS; lane 8, DNA incubated with wild type then digested with 0.5 U Dnase I before addition of SDS. (**c**) Mth10b, Sso10b and their single mutants: Lane 1, plasmid alone; lane 2, plasmid with addition of SDS; lane 3 to 10, DNA incubated with Mth10b, N11K, I39R, T77R, Sso10b, K16N, R44I and R83T then digested with 0.01 U Dnase I before addition of SDS.

A simple experiment was performed to test the ability of Mth10b, Sso10b and their variants to protect DNA against nuclease attack. As shown in Figure 7a, after treatment with Dnase I, the two bands corresponding to the positive control (lane 2) were still present in the sample of DNA incubated with wild type Sso10b (lane 8), whereas DNA incubated with the Sso10b triple mutant (lane 6) was degraded totally as was the control (lane 4). Accordingly, as shown in Figure 7b, DNA incubated with wild type Mth10b (lane 8) was degraded totally, whereas the Mth10b triple mutant could protect DNA against attack from Dnase I (lane 6). The protective ability of the Mth10b triple mutant seems a little lower than that of wild type Sso10b, indicating that the Mth10b triple mutant binds DNA more weakly than Sso10b. This may be due to electrostatic repulsion between the extremely negatively charged convex surface of Mth10b (Figure 3b, bottom left) and DNA. Accordingly, since the corresponding surface in Sso10b is neutral (Figure 3b, bottom right), there was no similar electrostatic repulsion between Sso10b and DNA. Moreover, the Mth10b single mutant I39R and two single mutants (K16N and R83T) of Sso10b of Sso10b also displayed the ability to protect DNA from Dnase I digestion to various extents (Figure 7c). These results suggest that typical Sac10b family proteins should be evenly distributed over the DNA without sequence specificity, and that the three positively-charged residues should play key roles in the interaction between typical Sac10b family proteins and DNA.

## Discussion

Typical Sac10b family proteins bind to DNA without sequence specificity, making it difficult to study the DNA-binding mechanism by traditional crystallization of a protein-DNA complex. In a recent study [23], we described atypical features of Mth10b from *M. thermoautotrophicum ΔH*, revealing that it is an acidic protein that binds to neither DNA nor RNA *in vitro*. In this work, we set out to clarify the structural basis of its unique characteristics and the molecular mechanism underlying the interactions of typical Sac10b family proteins with DNA.

We have determined the crystal structure of *M. thermoautotrophicum ΔH* Mth10b at a resolution of 2.2 Å (Figure 2). Its structure displays high similarity with its archaeal homologs (Figure 3a), but three pairs of conserved positively-charged residues located at the presumed DNA-binding surface are found to be substituted in Mth10b, suggesting that these positively-charged residues should play a key role in DNA-binding. To verify this hypothesis, through amino acids interchanges, four mutants of Mth10b and four mutants of Sso10b were constructed. Mth10b mutants N11K, I39R and T77R with the neutral residues changed to corresponding positively-charged residues acquired DNA-binding activity and the triple mutant N11KI39RT77R exhibited even higher DNA-binding activity. Conversely, removal of corresponding positively-charged residues in Sso10b greatly weakened its DNA-binding ability.

**Figure 8:**
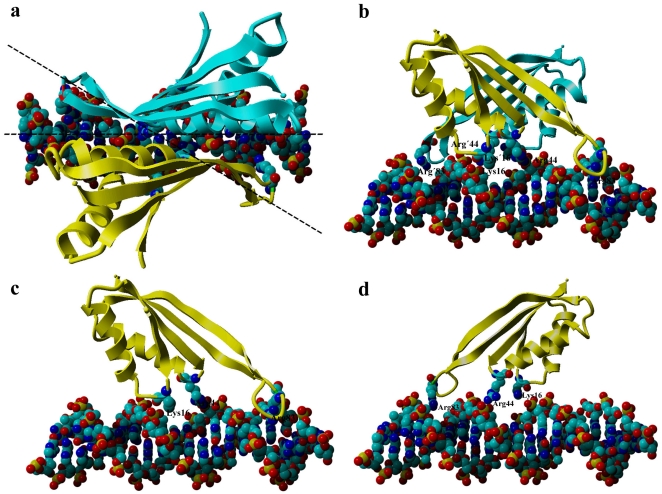
Model of the Sso10b dimer binding to DNA: (a) and (b) Orthogonal views showing how one Sso10b dimer might bind to DNA. (**c**) and (**d**) Stereo-views showing how one Sso10b monomer might bind to DNA. Sso10b is shown in a ribbon diagram, in which the three pairs of key positively-charged residues are highlighted. Residues with a prime (or without) belong to the same monomer. The DNA is depicted by ball models, in which phosphate groups are colored in red and yellow. The two dashed lines indicate the angle of about 30 degrees at which the long axis of the dimer threading the tips of the two β-hairpin arms intersects the long axis of the DNA duplex.

Pervious reports have shown that the concave surface of Sso10b is the DNA-binding surface and that Sso10b binds to DNA in its minor grooves [10], [14]. Based on these results and our new findings, we propose a new model that describes the mechanism of the interactions between typical Sac10b family proteins and DNA, using Sso10b as a prototype. Our results indicate that, in addition to Lys16 described previously [14], [17], [19]–[21], two further pairs of positively-charged residues, Arg44 and Arg83 in each monomer of Sso10b, play critical roles in DNA-binding. These residues are exactly located in the three regions indentified by Cui *et al*. by NMR [10], which are aligned in one plane and form a Latin Cross: two pairs of basic residues, Arg44 and Arg83 from each monomer, are located in a line and form the longer vertical bar, while the other pair of residues, Lys16 from the two monomers, is located at the center of the dimer concave, forming the shorter horizontal bar that appears to intersect the longer vertical bar perpendicularly (Figure 3c, right). The model proposed here is based on standard B-form DNA duplexes whose helical repeat is 10 bp. Unlike previous models [14], [17], [19]–[21], in our model, the long axis of the dimer threading the tips of the two β-hairpin arms is not parallel to the long axis of the DNA duplex, but intersects it at an angle of about 30 degrees, as indicated by the two dashed lines in Figure 8a. This angle is very important, and means that the two flexible β-hairpin arms (L5) of the dimer can enter into equivalent minor grooves without steric clashes. In the minor grooves, the two positively-charged residues (Arg83), located at the tips of the two L5 loops, grasp the DNA like two hands through electrostatic interactions with the phosphates. The highly positively-charged central body is placed between these flanking minor groove interactions making DNA contacts in the major groove regions. The basic diamond-shaped area of the central body, which is composed of the other two pairs of positively-charged residues (Lys16 and Arg44 from each monomer located at the tips of the L1 and L3 loops respectively), clutch the DNA through electrostatic interactions with the phosphates. In summary, the Sso10b dimer crosslocks the DNA duplex via the interaction of three pairs of positively-charged residues with the phosphates of the DNA (Figure 8). *In vivo*, its DNA affinity may be regulated by acetylation at Lys16.

We next considered how the dimers are organized on the DNA duplex. Using electron microscopy, an early report indicated that at low protein:DNA ratios, Sac10b envelops two strands of dsDNA to form a linear, flexible structure. Binding seems to be initiated at one or a few positions on DNA. Once such a complex has been initiated, it is elongated by addition of Sac10b molecules to already bound protein rather than by randomly binding to DNA [6]. When excess protein is added to pack all the DNA into the Sac10b-2×dsDNA complex, the two strands gradually open again and a new type of nucleoprotein is formed containing only one dsDNA [6]. Xue *et al.* also found that Ssh10b has two distinctively different modes of DNA-binding, depending on binding density [8]. In low-density binding mode, Ssh10b binds to 10–12 bp of DNA, whereas, in high-density binding mode, the protein appears to bind DNA at a much lower binding stoichiometry [8]. Based on our new model describing the interaction of Sso10b dimers with DNA (Figure 8) and our results on protein-DNA interactions, we delineated the molecular mechanism of dimer organization on the DNA duplex.

**Figure 9:**
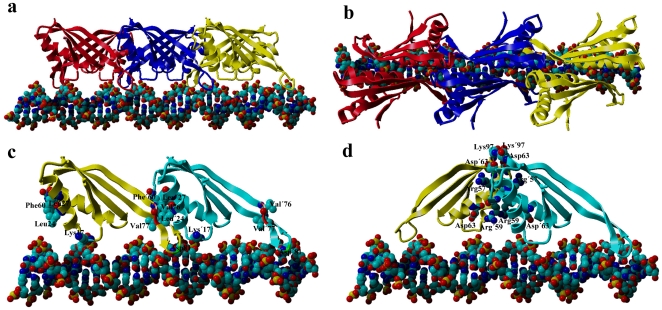
Low-binding density model. (**a**) and (**b**) Orthogonal views showing how Sso10b dimers might be organized in a head-to-tail fashion along the axis of the DNA duplex in the low-binding density mode. Some additional interactions may arise between two ipsilaterally adjacent monomers (**c**) and between two contralaterally adjacent monomers (**d**). The residues involved are highlighted. Residues with (or without) a prime belong to the same monomer.

In our model, as mentioned above, the long axis of the dimer intersects the DNA duplex at an angle of about 30° (Figure 8a). This angle allows for stacking of more dimers to the initiating dimer in a head-to-tail manner without steric clashes; one dimer covers about 15 bp of DNA, and the additional dimers along the long axis of the DNA only require 10 bp DNA (Figure 9a and 9b). Therefore, when Sso10b binds to DNA duplexes of sufficient length, the binding stoichiometry is about 10 bp per dimer, agreeing well with reported values in low-density binding mode [8] and our SPR results for the single mutant T77R (Figure 6a). However, on average, the DNA helical repeat of B-form DNA is about 10.4 bp under physiological conditions [26], indicating that dimers binding to the DNA duplex in this head-to-tail manner tighten the DNA “screw” slightly to 10 bp per turn in order to provide the same arrangement of binding sites along the long axis. In our opinion, this is the molecular basis of the formation of the Sac10b-2×dsDNA complex [6] and the fact that Sso10b can constrain negative DNA supercoils [8]–[10], since two tightened DNA double-helices tend to intertwine with each other. Some additional interactions may arise between two consecutive dimers in this head-to-tail manner. A hydrophobic interface may be formed between the Val76 and Val77 residues of one monomer and the Leu′24, Leu′27, and Phe′60 residues of the ipsilaterally adjacent monomer (Figure 9c). In addition, a salt bridge (Asp81–Lys′17) may be present between the two ipsilaterally adjacent monomers (Figure 9c). More complicated electrostatic interactions may be present between contralaterally adjacent monomers, involving two isolated salt bridges, Asp51–Arg′59 and Asp′51–Arg59, and two small ion-pair networks, Arg57–Asp′63–Lys97 and Arg′57–Asp63–Lys′97 (Figure 9d). In summary, 12 residues from each monomer, Lys17, Leu24, Leu27, Asp51, Arg57, Arg59, Phe60, Asp63, Val76, Val77, Asp81 and Lys97, almost all conserved in the Sac10b family, are involved in the interactions between two adjacent dimers (Figure 1). These interactions play a role in orientating new binding dimers and are the main molecular basis underlying the cooperative binding of typical Sac10b members to DNA, and can explain why dimers bind to DNA in this head-to-tail fashion at low-binding density. This is in line with previously published mutagenesis experiments: mutagenesis of Lys17 and Phe60 in Sso10b resulted in a several-fold reduction in DNA-binding affinity [13], [16]; mutagenesis of Lys11, Leu18 and Phe54 in Afu10b (corresponding to Lys17, Leu24 and Phe60 in Sso10b respectively) also resulted in a several-fold reduction in DNA-binding affinity [20].

**Figure 10:**
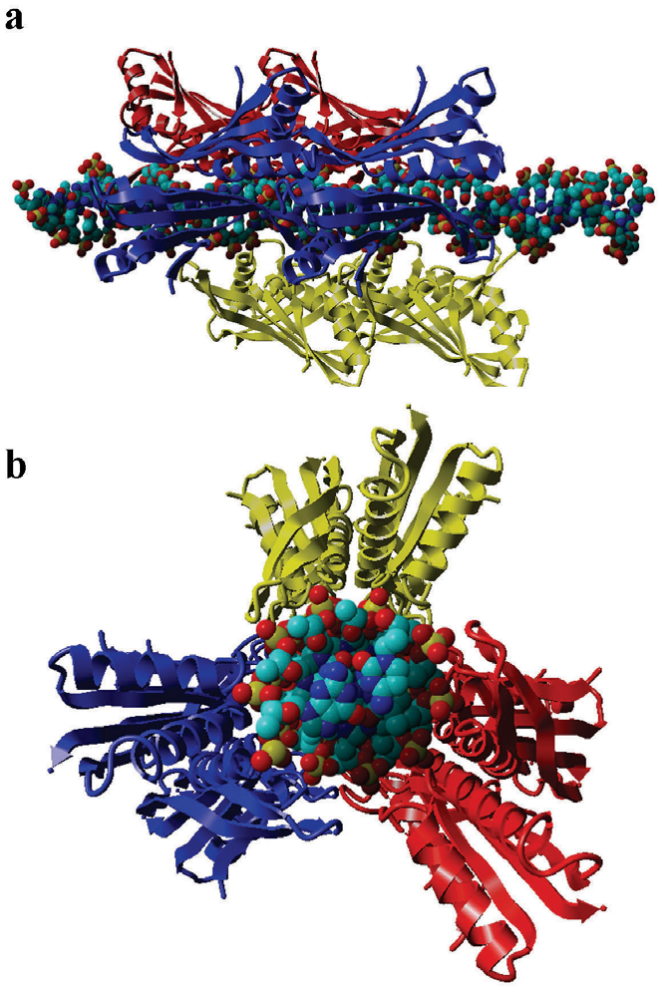
High-binding density model. (**a**) and (**b**) Stereo-views showing how Sso10b dimers might be positioned side-to-side along the axis of the DNA duplex in the high-binding density mode. Three columns of dimers, rotated by ∼120° with respect to one another, bind along the axis of the DNA duplex. The DNA duplex is packed with three columns of Sso10b dimers like a cable made up of a DNA cable core and an Sso10b cable sheath.

SPR measurements of the triple mutant of Mth10b suggested a saturation binding stoichiometry of about 3.3 bp per dimer, indicative of the formation of a different complex involving more proteins. When protein concentration is high, since more dimers will tend to bind to the DNA, the two DNA double-helices that are intertwined at low-binding density are separated, and three columns of dimers, rotated by ∼120° with respect to one another to avoid steric clashes, bind along the axis of the DNA duplex (Figure 10). The dimers of these three columns all bind to the DNA duplex in the head-to-tail manner described above. Two salt bridges, Asp81–Lys′17 and Lys17–Asp′81, may be present between two side-by-side adjacent dimers. The three columns of Sso10b dimers pack tightly around the DNA duplex like the sheath of a cable (Figure 10), which could explain the resistance of DNA, to which Sac10b family proteins are bound, to Dnase I digestion described in our work (Figure 7) as well as in an early report [6].

In the case of other typical Sac10b family members, since the lengths of the flexible β-hairpin arms vary, the length of the DNA covered by each dimer and the angle at which they intersect the long axis of the DNA may also be slightly different. However, their DNA-binding mechanisms should be similar with that of Sso10b presented here. Taken together, we have identified the critical amino acid residues that are involved in DNA-binding of Sac10b family proteins, and delineated the molecular mechanism underlying the interaction of Sac10b family proteins with DNA.

What, then, is the exact function of Mth10b *in vivo*? Although Mth10b has no ability to interact with nucleic acids *in vitro*, the overall structure of Mth10b still closely resembles other Sac10b family DNA-binding proteins. We hypothesize that Mth10b may be recruited by a given regulation factor through electrostatic interactions formed between its extremely negatively charged convex surface and the positively-charged concave surface of the regulation factor to participate in gene expression and regulation. Experiments to clarify the exact function of Mth10b are currently in progress.

## Materials and Methods

### Materials

The plasmid pET11a from Novagen was used to make the vector-DNA construct. Expression plasmids pET11a-*mth10b* containing the *mth10b* gene [23] and pET11a-*sso10b* containing the *sso10b* gene [11] were obtained from our laboratory stocks. *Escherichia coli* DH5α and BL21 (DE3) cells were used for plasmid cloning and protein expression, respectively. Enzymes and reagents for DNA manipulations were purchased from TAKARA. Plasmid Miniprep Kits and Gel Extraction Kits were obtained from OMEGA. Yeast extract and Tryptone were purchased from OXOID. Isopropyl β-D-thiogalactoside (IPTG) was obtained from MERCK. All chemicals were of analytical grade for biochemical use. All apparatus and chromatography materials were purchased from GE.

### Crystallization and structure determination

Purified Mth10b in a buffer containing 10 mM HEPES, pH 7.2, 150 mM NaCl was concentrated to ∼10 mg/ml for the initial screening of crystals. Crystals were grown by the hanging drop vapor diffusion method at 4°C for about 3 days, using an equal volume (0.5 µl) of protein and crystallization buffer over a 0.5 ml reservoir composed of 0.1 M NaCl, 1.6 M (NH_4_)_2_SO_4_, 0.1 M HEPES, pH 7.5. A 2.2 Å dataset was collected on beamline BL17U at the Shanghai Synchrotron Research Facility (SSRF) and processed with HKL2000 [27]. The structure was determined by molecular replacement with Sso10b (PDB code: 1H0X) as the search model in program PHASER [28]. Refinement and model building were conducted using COOT [29] and PHENIX [30], respectively. Statistics for data collection and refinement are given in Table 1.

**Table 1:** Crystallography statistics.

Data collection	
Beamline	SSRF BL17U
Wavelength	0.9793
Space group	I222
Cell dimensions	
a, b, c (Å)	52.90, 76.26, 87.50
α, β, γ (°)	90, 90, 90
Resolution (Å)	50-2.20 (2.26-2.20)
R_merge_ (%)	12.6 (36.8)
I/σ(I)	14.7 (2.1)
Completeness (%)	98.9 (91.4)
Redundancy	5.6 (3.8)
Refinement	
Resolution (Å)	28.7–2.20
No. Reflections	9122
R_work_/R_free_ (%)	21.3/27.9
No. atoms	
Protein	1327
Water	15
B-factors (Å2)	
Protein	54.9
Water	52.5
R.m.s. deviations	
Bond lengths (Å)	0.006
Bond angles (°)	0.900
Ramachandran plot	
Most favored	91.4
Allowed	7.2
Generally allowed	0.7
Disallowed	0.7

### Mutant protein expression and purification

All expression plasmids of Mth10b variants and Sso10b variants were constructed from the parental plasmids pET11a-*mth10b* and pET11a-*sso10b*. All mutations were introduced by site-directed mutagenesis through overlap extension PCR. The construct for each variant was verified by DNA sequencing. Recombinant proteins in this work were all expressed in *Escherichia coli* BL21 (DE3) and purified as described previously [23], [24].

### Biosensor-SPR analysis

DNA-protein interactions were analyzed by SPR using a BIAcore 3000 system (GE). To create a non-specific DNA duplex for SPR experiments, the N-terminal 110 bp fragment of the *mth10b* gene (MTH1483) was amplified from the plasmid pET11a-*mth10b* by PCR with the forward primer 5′- gTgATgTCAgAggAgAATgTAgTATAC -3′ and the reverse primer 5′- gCTTTAAggATCACTTCACTggTC -3′. The forward primer was labeled with 5′-biotin. The PCR product was purified from a 1% agarose gel using a Gel Extraction Kit (OMEGA). The extracted DNA duplex was diluted about 10-fold in basic running buffer (20 mM Tris, 1 mM EDTA, 0.005% Tween20/pH 7.5) and applied to the streptavidin-coupled sensor chip (SA chip) surface at 10 µL/min to generate about 75 response units (76.4 RU). Measurements were performed in basic running buffer containing different concentrations of NaCl at 25°C. Protein samples were prepared by serial dilution with running buffer from the stock solution and applied to the sensor surface at a flow rate of 30 µL/min for 1–2 min. Postinjection disassociation was monitored in running buffer at the same rate for 2 min. Surface regeneration between injections was achieved using a 1-min injection of basic running buffer containing 0.5 M NaCl. All experiments were performed in triplicate and any background signal from the reference flow cell without immobilized ligand was substracted from all data sets. Sensorgrams, RU versus time, at different concentrations, for the binding of each protein to DNA were obtained, and the RU in the steady-state region was determined by linear averaging over a selected time span. When there was no obvious cooperativity, binding parameters (the maximal binding capacity (R_max_) and the apparent K_D_) were obtained by data analysis with a simple 1∶1 Langmuir interaction model using BIAevaluation software. When there was obvious cooperativity, R_max_ was estimated from the binding curves directly, and the apparent K_D_ was estimated from the concentration of the protein required to bind with 50% saturation. The saturation binding stoichiometries of tested proteins were calculated according to Rmax values and the amount of DNA fixed on the chip.

### Analysis of protecting DNA from Dnase I digestion

In order to test whether or not the DNA was protected from nuclease attack by the tested proteins, a simple experiment was designed. Approximately 400 ng plasmid pAS22 (3789 bp) was incubated with 10 µg recombinant protein in binding buffer (20 mM Tris/pH 7.5) in a total volume of 15 µl at room temperature for 20 min. After addition of Dnase I (TAKARA), the incubation was continued at room temperature for another 20 min. SDS was then added to a final concentration of 1% (W/V) to disable the nuclease and disassemble the DNA-protein complex. Samples were resolved by electrophoresis in 1.5% agarose gels in 1×TAE buffer at constant voltage. After electrophoresis, the gels were stained with ethidium bromide and visualized under UV light. Several controls were performed parallely.
